# Use of recombinant activated factor VII to stop post-kidney biopsy bleeding in a child

**DOI:** 10.1007/s00467-025-07063-y

**Published:** 2025-12-02

**Authors:** Elena Park, Stephanie P. Kerkvliet, Andrew D. Hughes, Amy J. Kogon

**Affiliations:** 1 Geisinger College of Health Sciences, Scranton, PA USA; 2 Division of Pediatric Nephrology, University of Pennsylvania, Philadelphia, PA USA; 3 Division of Hematology, Children’s Hospital of Philadelphia, Philadelphia, PA USA

**Keywords:** Recombinant activated factor VII, Kidney biopsy, Complication, Hemorrhage, Case report

## Abstract

Kidney biopsy is the standard of diagnosis for many kidney diseases and conditions. Severe bleeding following a kidney biopsy is a complication for which limited therapeutic interventions exist. The use of recombinant activated factor VII (rFVIIa) is described in pediatric patients to control severe intra-operative hemorrhage and has been used in at least one adult patient to manage post-kidney biopsy hemorrhage. We present a 10-year-old female who presented with acute kidney injury, thrombocytopenia, and hypertension, ultimately diagnosed with ANCA-vasculitis. Following a kidney biopsy, the patient experienced a large, expanding hematoma with active bleeding that did not improve despite pre-biopsy desmopressin, 90 min of pressure to the biopsy site, and platelet transfusions. Recombinant activated FVIIa was administered, and the bleeding immediately stopped. This case supports the use of recombinant FVIIa in post-kidney biopsy hemorrhage when bleeding is not controlled using conventional strategies.

**Supplementary information:**

The online version contains supplementary material available at 10.1007/s00467-025-07063-y.

## Introduction

Kidney biopsy is the gold standard to diagnose and guide treatments of many kidney and autoimmune diseases [1]. Percutaneous kidney biopsy is generally well tolerated; self-limited bleeding is a common complication, but the risk for significant hemorrhage is < 1% [1]. Bleeding risk can be mitigated prior to renal biopsy by stopping anticoagulation and antiplatelet therapy, treating hypertension and thrombocytopenia and administering desmopressin (DDAVP) to patients with uremia, thrombocytopenia, or platelet dysfunction [1]. During kidney biopsies, bleeding risk is minimized by localizing the kidney with ultrasound guidance, the use of spring-loaded biopsy needles, and applying directed pressure over the biopsy site immediately following sampling [1]. In severe cases, kidney vascular embolization or surgical interventions may be required to stop bleeding; however, there are limited reports of non-interventional treatments to address post-kidney biopsy hemorrhage [1].

**Fig. 1:**
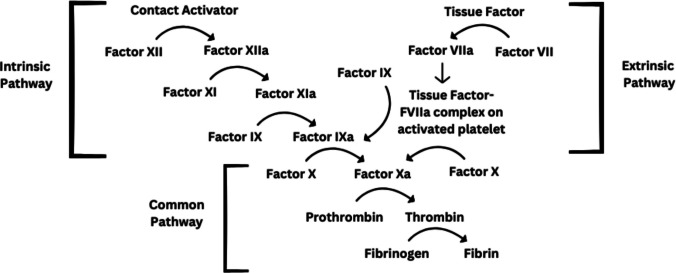
The coagulation cascade demonstrating the role of tissue factor–FVIIa complex

Factor VII and tissue factor make up the extrinsic pathway of the coagulation cascade and activate factor X, to ultimately generate thrombin and fibrin [2] (Fig. 1). The extrinsic pathway is a critical step for the initiation of clot formation in response to vascular injury, as evidenced by the severe, hemophilia-like phenotype seen in patients who are deficient in factor VII [2]. The rate and amount of thrombin generation positively correlate with the number of platelets present [2]. Therefore, in patients with thrombocytopenia, a smaller number of platelets may adhere to a site of injury, leading to a less stable initial platelet plug and less surface area on which thrombin can form [2].

Recombinant activated factor VII (rFVIIa) is FDA approved for bleeding in patients with severe bleeding disorders including hemophilia [3]. rFVIIa binds specifically to the surface of activated platelets inducing activation of downstream factors and thrombin generation [2]. Widespread thrombosis is avoided because rFVIIa binds to activated platelets, localizing its effects to sites of bleeding [2, 4]. The effectiveness of rFVIIa has led to more generalized use, particularly during refractory, intraoperative bleeding [4]. Here, following the recommendations of the CARE Checklist for writing a case report (Supplement), we present a pediatric case of severe hemorrhage following a kidney biopsy treated with rFVIIa.

## Case report

A previously healthy 10-year-old female presented with four days of diarrhea, vomiting, epistaxis, and headache without oliguria, gross hematuria, or edema. Initial evaluation revealed hypertension (158/126), respiratory distress, hyperkalemia (K 5.6 mmol/L), acidosis (CO2 < 5 mmol/L), azotemia (BUN 146 mg/dL), acute kidney injury (Cr 17 mg/dL), normocytic anemia (hemoglobin 4.3 g/dL), and extensive bilateral pulmonary infiltrates on chest X-ray (Table 1). She was admitted to the pediatric intensive care unit, where she was intubated and noted to have blood-tinged secretions concerning for pulmonary hemorrhage. She was treated with continuous kidney replacement therapy (CKRT), nicardipine, and red blood cell transfusion. ANCA titers were sent, but results would not be available until 4 days later.

**Table 1:** Blood pressure and laboratory data at presentation and preceding kidney biopsy

Data	Presentation	Preceding biopsy	Reference
Blood pressure (mmHg)	158/126	105/67	116/76 (95th%ile)
Potassium (mmol/L)	5.6	3.3	3.8–5.4
Bicarbonate (mmol/L)	< 5	21	20–26
BUN (mg/dL)	146	77	5–17
Creatinine (mg/dL)	17.12	6.90	0.20–0.50
Hemoglobin (g/dL)	4.3	11.0	11.5–15.5
Platelets (•10^3^/dL)	230	116	150–450
INR	1.5	1.48	< 3.00
PT (secs)	18.6	18.4	11.1–13.4
PTT (secs)	28.5	31.8	25.0–37.0
Complement C3 (mg/dL)	77		90–187
Complement C4 (mg/dL)	24.8		16.0–45.0

One day following admission, she showed evidence of clinical and laboratory improvement with normal electrolytes and improved BUN (mg/dL), hemoglobin (11 g/dL), and blood pressure on nicardipine (Table 1). Highest on the differential was antineutrophil cytoplasmic antibodies (ANCA) vasculitis, followed by other immune-related glomerulopathies. Given the patient’s stabilization and the need for a confirmatory diagnosis to guide specific therapy, she underwent a renal biopsy on the second day of hospitalization. To minimize the risk of bleeding, she received vitamin K (5 mg) for prolonged prothrombin time (PT) and DDAVP (0.3 mcg/kg) for uremic platelet dysfunction prior to the biopsy. Blood pressures remained in the normal range with nicardipine drip during the procedure. Using ultrasound, the lower pole of the left kidney was identified, and three needle passes were made with a 16-gauge, spring-loaded biopsy needle.

**Fig. 2:**
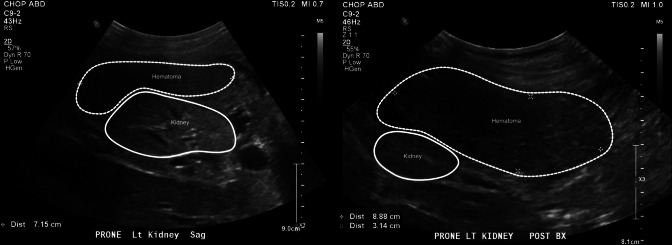
Post-renal biopsy renal ultrasound images of the kidney and hematoma

Following the final pass, the ultrasound showed a hematoma with active bleeding. Pressure with the providers’ hands was immediately applied over the left flank. Bleeding was monitored by serial ultrasound approximately every 5–10 min, which showed an expanding hematoma, up to a maximum of 9.1 × 3.2 × 5.7 cm (Fig. 2). An additional dose of DDAVP was administered (0.3 mcg/kg), along with fresh frozen plasma and platelets in attempts to reduce the bleeding. Her PT improved to 15.9 s and her platelet count was 108•10^3^/dL, but the bleeding persisted. Interventional radiology was urgently consulted, and plans were made for a possible embolization procedure. With ongoing bleeding following two red blood cell transfusions, the team administered NovoSeven (90 mcg/kg), a rFVIIa medication. Immediately after administration, the bleeding stopped, and no additional medical or procedural interventions were required. The PT normalized to 8.5 s. She was initiated on pulse steroids (methylprednisolone 30 mg/kg/day) for 3 days immediately following the biopsy as initial treatment for glomerulonephritis while awaiting the biopsy results.

Biopsy results reviewed the day following the biopsy demonstrated a pauci-immune crescentic glomerulonephritis with chronic features. Disease-targeting treatments including rituximab (750 mg/m^2^) and plasmapheresis (seven treatments) were initiated. ANCA myeloperoxidase (MPO) IgG returned 3 days after the biopsy was elevated (38 AU/mL), all consistent with the diagnosis of p-ANCA vasculitis. She continued to have intermittent blood-tinged secretions from her endotracheal tube without additional bleeding complications. Follow-up ultrasound demonstrated decreasing size of the perinephric hematoma 1 day after the biopsy. She was successfully extubated 2 days following her kidney biopsy. She had no significant thrombotic complications, though she developed an occlusive superficial thrombosis/thrombophlebitis in her right cephalic vein 11 days after the biopsy that was treated conservatively. Ultimately, the patient transitioned to intermittent hemodialysis and was discharged after 3 weeks to receive outpatient hemodialysis. Her kidney function never recovered and she received a kidney transplant approximately 1 year following initial presentation.

## Discussion

To our knowledge, the above case represents the first described use of rFVIIa to successfully stop post-kidney biopsy hemorrhage in a pediatric patient. Obtaining kidney tissue was essential to diagnosing and treating our patient. While there was strong clinical suspicion for ANCA-associated vasculitis (AAV) based on the initial presentation, her C3 was low, which is atypical for this disease. A broader differential diagnosis included systemic lupus erythematosus (SLE), anti-glomerular basement membrane (GBM) disease, post-infectious glomerulonephritis, C3 glomerulopathy, hemolytic uremic syndrome (HUS), and cryoglobulinemic vasculitis. A kidney biopsy was required to provide a tissue diagnosis and guide targeted treatment. In particular, the use of rituximab as initial therapy is most specific to the treatment of AAV.

Practitioners must always carefully weigh the risks and benefits of kidney biopsy and delay biopsy in high-risk scenarios. In consideration of our patient’s improving clinical and laboratory status, anticipated timing of pending diagnostic laboratory results, and anticipated extubation, we believed the benefit to outweigh the risk, particularly with precautions, including administration of antihypertensive medications, vitamin K, and DDAVP prior to the procedure [1]. When standard measures to stop post-procedural bleeding were unsuccessful, the use of rFVIIa very quickly stopped the bleeding and prevented the need for embolization. We did not observe any negative effects of rFVIIa—the half-life of NovoSeven is a few hours, so the superficial thrombosis several days later is unlikely related [3]. It is notable that the PT prior to receiving rFVIIa was prolonged at 15.9, suggesting some degree of factor VII consumption; however, it is unlikely that this is the entire etiology of the patient’s bleeding. rFVIIa administration is expected to be effective even in the setting of normal endogenous FVII levels.

Based on our experience, the off-label use of rFVIIa in severe post-kidney biopsy hemorrhage should be considered when standard practices to prevent and stop bleeding are unsuccessful. A prior case described effective use of rFVIIa to stop post-kidney biopsy bleeding in an adult patient [5]. The success of rFVIIa treatment in this case report supports consideration of rFVIIa administration in events of post-kidney biopsy hemorrhage among pediatric patients [4, 5]. The use of rFVIIa is also supported by studies demonstrating safe use for intraoperative bleeding. When using rFVIIa, practitioners must weigh the risk of undesired thromboembolic events, though none was observed in our patient.

## Conclusion

Significant hemorrhage following a renal biopsy is uncommon, but when it occurs, using FVIIa as a non-invasive therapy to stop bleeding can be considered.

## **Summary**

### **What is new?**

- In the setting of post-kidney biopsy hemorrhage in a pediatric patient, off-label administration of recombinant activated factor VII effectively stopped bleeding.

## Supplementary information

Below is the link to the electronic supplementary material.

ESM 1 (PDF 711 KB)

## Data Availability

Data sharing not applicable to this article as no datasets were generated or analyzed during the current study.
