# Supplementary material for: Use of recombinant activated factor VII to stop post-kidney biopsy bleeding in a child
Source: Pediatr Nephrol. 2025 Dec 2;41(4):1007–11. doi: 10.1007/s00467-025-07063-y (PMC12953263; doi:10.1007/s00467-025-07063-y)
Supplement: Supplementary file 1 — (PDF 711 KB) [file 467_2025_7063_MOESM1_ESM.pdf]

CARE case report guidelines

CARE Checklist of information to include when writing a case report

(cc) BY-NC-ND

Topic	Item	Checklist item description	Reported on Line
Title	1	The diagnosis or intervention of primary focus followed by the words "case report"	Title
Key Words	2	2 to 5 key words that identify diagnoses or interventions in this case report, including "case report"	line 37
Abstract (no references)	3a	Introduction: What is unique about this case and what does it add to the scientific literature?	63-74
	3b	Main symptoms and/or important clinical findings	76-84
	3c	The main diagnoses, therapeutic interventions, and outcomes	95-114
	3d	Conclusion—What is the main "take-away" lesson(s) from this case?	135-136
Introduction	4	One or two paragraphs summarizing why this case is unique (**may include references**)	116-125
Patient Information	5a	De-identified patient specific information.	76-77
	5b	Primary concerns and symptoms of the patient.	76-84
	5c	Medical, family, and psycho-social history including relevant genetic information	76-84
	5d	Relevant past interventions with outcomes	94-103
Clinical Findings	6	Describe significant physical examination (PE) and important clinical findings.	76-114
Timeline	7	Historical and current information from this episode of care organized as a timeline	76-114
Diagnostic Assessment	8a	Diagnostic testing (such as PE, laboratory testing, imaging, surveys).	94-103
	8b	Diagnostic challenges (such as access to testing, financial, or cultural)	n/a
	8c	Diagnosis (including other diagnoses considered)	99-100
	8d	Prognosis (such as staging in oncology) where applicable	n/a
Therapeutic Intervention	9a	Types of therapeutic intervention (such as pharmacologic, surgical, preventive, self-care)	102
	9b	Administration of therapeutic intervention (such as dosage, strength, duration)	102
	9c	Changes in therapeutic intervention (with rationale)	n/a
Follow-up and Outcomes	10a	Clinician and patient-assessed outcomes (if available)	104-114
	10b	Important follow-up diagnostic and other test results	104-114
	10c	Intervention adherence and tolerability (How was this assessed?)	110-112
	10d	Adverse and unanticipated events	110-113
Discussion	11a	A scientific discussion of the strengths AND limitations associated with this case report	122-139
	11b	Discussion of the relevant medical literature **with references**.	117-139
	11c	The scientific rationale for any conclusions (including assessment of possible causes)	131-139
	11d	The primary "take-away" lessons of this case report (without references) in a one paragraph conclusion	137-139
Patient Perspective	12	The patient should share their perspective in one to two paragraphs on the treatment(s) they received	n/a
Informed Consent	13	Did the patient give informed consent? Please provide if requested ... not requested	Yes ☐ No ☐
